# *In Vivo* Determination of Mouse Olfactory Mucus Cation Concentrations in Normal and Inflammatory States

**DOI:** 10.1371/journal.pone.0039600

**Published:** 2012-07-20

**Authors:** Senthil Selvaraj, Kevin Liu, Alan M. Robinson, Victoria A. Epstein, David B. Conley, Robert C. Kern, Claus-Peter Richter

**Affiliations:** 1 Department of Otolaryngology-Head and Neck Surgery, Feinberg School of Medicine, Northwestern University, Chicago, Illinois, United States of America; 2 Department of Biomedical Engineering, Northwestern University, Evanston, Illinois, United States of America; 3 The Hugh Knowles Center, Department of Communication Sciences and Disorders, Northwestern University, Evanston, Illinois, United States of America; University of Tokyo, Japan

## Abstract

**Objective:**

Olfaction is impaired in chronic rhinosinusitis (CRS). The study has two aims: (1) to determine whether changes in cation concentration occur in the olfactory mucus of mice with CRS, which may affect chemo-electrical transduction, (2) and to examine whether these alterations are physiologically significant in humans.

**Study Design:**

Animal study in mice and translational study in humans.

**Methods:**

Inflammation was induced by sensitization and chronic exposure of 16 C57BL/6 mice to *Aspergillus fumigatus*. The control group included 16 untreated mice. Ion-selective microelectrodes were used to measure free cation concentrations in the olfactory mucus of 8 mice from each treatment group, while the remaining mice were sacrificed for histology. To validate the findings in the animal model, olfactory threshold was measured in 11 healthy human participants using *Sniffin’ Sticks* before and after nasal irrigation with solutions that were composed of either of the cation concentrations.

**Results:**

In 8 mice, olfactory mucus of chronically inflamed mice had lower [Na^+^] (84.8±4.45 mM versus 93.73±3.06 mM, p = 0.02), and higher [K^+^] (7.2±0.65 mM versus 5.7±0.20 mM, p = 0.04) than controls. No difference existed in [Ca^2+^] (0.50±0.12 mM versus 0.54±0.06 mM, p = 0.39). In humans, rinsing with solutions replicating ion concentrations of the mouse mucosa with chronic inflammation caused a significant elevation in the median olfactory threshold (9.0 to 4.8, p = 0.003) but not with the control solution (8.3 to 7.8, p = 0.75).

**Conclusion:**

Chronic inflammation elevates potassium and lowers sodium ion concentration in mice olfactory mucus. Nasal irrigation with a corresponding solution induced olfactory threshold shift in humans.

## Introduction

Chronic rhinitis and rhinosinusitis affect approximately 33 million people in the United States and are primary etiologies for olfactory loss among patients [1]. Our understanding of the pathogenesis in olfactory function is quite limited. The mechanisms by which rhinitis and rhinosinusitis cause olfactory dysfunction are likely multifactorial and include direct effects of inflammatory mediators on olfactory sensory neurons (OSNs) in addition to respiratory mucosal edema and reduced airflow to the olfactory cleft [2]. One result of inflammation may be a change in the ion concentration of the olfactory mucus, which could potentially interfere with the OSN chemo-electrical signal transduction process and olfaction.

The olfactory cleft is rich in OSNs within the olfactory epithelium that project sensory cilia into the nasal cavity and are bathed in a specialized mucus layer. These cilia are rich in membrane-bound G-protein coupled odorant-binding receptor proteins [3]. Odorant binding to these receptor proteins activates G_olf_, a G protein expressed in OSNs that primarily activates adenylyl cyclase type III, thereby raising the intracellular cyclic-AMP concentration [4]. This triggers opening of the olfactory specific trans-membrane olfactory cyclic nucleotide-gated channels (OCNC) that are permeable to both monovalent and divalent cations, allowing mainly influx of Na^+^ and Ca^2+^ from the mucus into the ciliary cytoplasm. The ion influx depolarizes the OSN and triggers an action potential. Furthermore, calcium may amplify the signal by binding to and opening Ca^2+^ activated Cl^−^ channels, which further depolarizes the cell [5]. The action potential is transmitted to the brain via secondary synapses in the olfactory bulb and interpreted as an odor [3], [6], [7].

The dynamics of OCNCs have been extensively studied [3], [6], [8]. For example, it has been demonstrated that the presence of K^+^ and Na^+^ ions modulate the open probability of the channels, not only by altering the lifetime in the fully open state, but also by affecting the channel conductance [9]–[11]. Patch-clamping experiments have indeed demonstrated that lowering the mucosal sodium concentration in frogs induces a dramatic decrease in neural activity of OSNs [9]. Consequently, changes in mucosal cation concentrations can alter sensitivity to odorant stimulation [12].

The ionic composition of nasal airway mucus has been measured in rodents and humans, using both microdialysis and X-ray microanalysis techniques [13], [14]. Olfactory mucus ion composition data are available for frogs, measured using a spectrophotometry technique [15]. Our study employed ion-selective microelectrodes to measure and compare the *in vivo* free sodium, potassium and calcium concentrations of olfactory mucus between control mice and mice with chronic nasal inflammation. We subsequently sought to determine the physiological significance of cation shifts in the human olfactory mucosa that resulted from chronic rhinosinusitis in mice by measuring olfactory thresholds before and after nasal irrigation with solutions replicating ion concentrations determined in the animal model.

**Table 1:** Composition of each cation selective microelectrode and concentrations of aqueous calibration solutions.

	Ionophore	Aqueous filling solution	Aqueous calibration solutions
**Na^+^**	Fluka- Sodium Ionophore II- Cocktail A	500 mM NaCl	500, 250, 125, 62.5, 31.3 mM NaCl
**K^+^**	Fluka- Potassium Ionophore I- Cocktail B	100 mM KCl	100, 50, 25, 12.5, 6.3, 3.1 mM KCl
**Ca ^2+^**	Fluka- Calcium Ionophore I- Cocktail A	100 mM CaCl_2_	100, 10, 1, 0.1, 0.01 mM CaCl_2_(CALBUF-1, World Precision Instruments)

## Materials and Methods

### Animals

Eight week-old C57BL/6 mice were housed in pathogen-free conditions at Northwestern University. Animal procedures were performed in accordance with guidelines of the National Institutes of Health and were approved by the Northwestern University Animal Use and Care Committee (Approval number: 2005-0882).

### Induction of Chronic Inflammation

A fungal protein extract of *A. fumigatus* (Strain ATCC 1022, Greer Laboratories, NC) was used to induce chronic rhinitis in mice [16]. Sixteen awake mice were sensitized to the allergen by repeated (3 times a week on consecutive days for 3 weeks) nasal aspiration of 100 µg of extract in 50 µL of normal saline solution, instilling 25 µL in each naris. A period of 14 days of no treatment followed to allow for the development of a humoral immune response. Animals were subsequently challenged with daily intranasal instillation of 100 µg of extract for 2 weeks. Eight mice were randomly selected for olfactory mucus ion measurements. The remaining 8 animals were sacrificed for histology 12–18 hours after the last administration of the antigen. A control group of 8 mice underwent the same protocol using normal saline only in place of the fungal extract while an additional 8 mice were also sacrificed for histology.

### Histology and Immunohistochemistry

Mouse heads were processed for immunohistochemistry as previously described [17]. Anesthetized mice underwent cardiac perfusion and fixation with cold 4% paraformaldehyde in 0.14 M, pH 7.4 phosphate buffered saline (PBS) and overnight fixation after excess tissue was removed. Heads were washed in PBS at room temperature for 2 hours and decalcified for 24 hours in a 4% formic acid and 2% sodium citrate solution at 4°C. Whole heads were dehydrated in graded ethanols, cleared in xylene, and embedded in paraffin wax. Coronal sections (7 µm) were cut and mounted on glass slides for histology. To assist in limiting analyses to the olfactory epithelium, differentiation from respiratory mucosa was achieved by immunohistochemical detection of olfactory marker protein (OMP), a small protein that is a specific marker for mature OSN cell bodies in the olfactory epithelium, their nerve bundles, and synapses in the olfactory bulb, but absent from respiratory mucosa [18].

Immunohistochemistry to detect Major Basic Protein (MBP) as a marker for eosinophil degranulation was performed as follows. Tissue sections on glass slides were de-paraffinized, rehydrated to water, treated with peroxidase and non-specific binding blocked with Mouse on Mouse (M.O.M.) reagent (Vector Laboratories Inc., Burlingame, CA) and incubated 1 hour at 37°C with Mouse anti-Human & Mouse Eosinophil Major Basic Protein (US Biological, Swampscott, MA) at 1∶200 dilution. Immunodetection was performed using mouse Vectastain ABC elite kit as per instructions and a three-minute reaction with 3–3′ diaminobenzamidine (DAB) substrate was used to develop a brown precipitate. Sections were dehydrated and glass coverslips mounted. Corresponding controls were included.

Eosinophilic inflammation was quantified both in the respiratory and olfactory epithelium. Eosinophils were counted in a 1000x field in 6 randomly assigned, corresponding locations using Sirius Red (3 hours) and hematoxylin stained coronal sections corresponding to regions identified as being olfactory epithelium by OMP immunohistochemistry (3 locations) and respiratory epithelium (3 locations). Sirius Red, a stain with great specificity for eosinophils, has been shown to enable greater ease in eosinophil identification through its excellent tissue contrast [19]. Eight chronically inflamed mice and 8 untreated mice were evaluated. For each animal, the eosinophils in both the olfactory and respiratory epithelium were counted twice. Those two numbers were averaged. The average for each animal was then used to calculate the mean of each treatment group.

### Surgery and Ion Concentration Measurements

#### Ion selective microelectrodes

A standard procedure to construct and calibrate the ion selective electrodes was used, which was previously established in the laboratory [20]. Micropipettes with a 3–6 µm tip diameter were pulled from borosilicate theta-glass capillaries (O.D. 1.5 mm) using a Flaming/Brown horizontal Micropipette Puller (Model P-97, Sutter Instr. Co.). Surface charges on the glass were neutralized by silanizing. Only 1 of the 2 barrels of each micropipette was filled with the ionophore cocktail and an aqueous backfilling solution, as listed in Table 1.

Voltages between the ion selective electrode and the reference electrode were measured with a Dual Microelectrode Amplifier VF102/C (Biologic, Claix, France). Ion concentrations were determined from the calibration curves for the individual electrodes.

A fresh electrode was used for each animal and each was calibrated before and after the *in vivo* measurements to ensure that they had not been damaged by the penetration through the olfactory epithelium. Pure solutions containing the test ions (sodium, potassium, or calcium) were used for calibration of the corresponding electrodes (see Table 1). The mean sensitivities in mV/decade were for Na^+^66.0±1.46 (N = 16), for K^+^65.1±1.32 (N = 16), and for Ca ^2+^30.0±0.81 (N = 16). For the *in vivo* measurements, an indifferent electrode was placed on the animals’ anterior skull bone.

#### Surgery

Mice were anesthetized with an intraperitoneal injection of Ketamine/Xylazine (120/10 mg/kg bodyweight). Skin and subcutaneous tissues were removed to gain access to the nasal bones. A dental drill with a 1 mm bit was used to remove a small ∼1 mm^2^ portion of the bone. Care was taken not to damage the underlying tissue. Mice were selected for surgery in a randomized and blinded manner.

**Figure 1:**
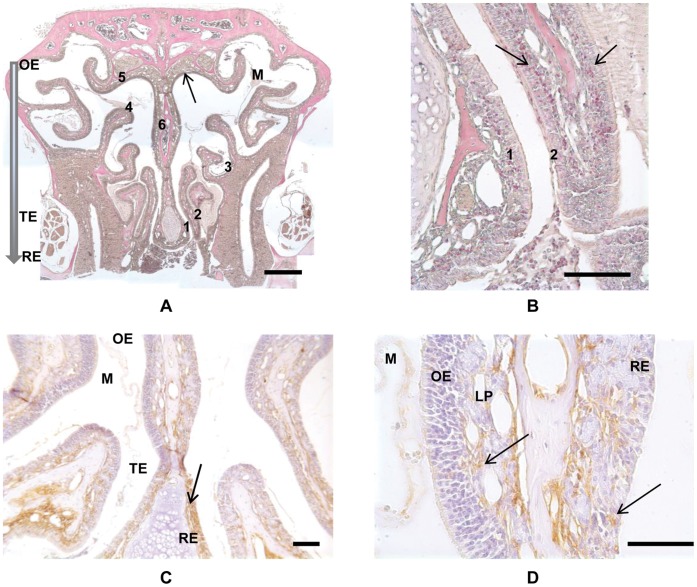
Demonstration of eosinophils and major basic protein (MBP) in a chronic allergen exposed mouse. A) Low power image of Sirius Red and hematoxylin stained coronal section of a chronic allergen exposed mouse. Numerals identify the six sampling areas, where specific 100 micron square areas were designated for eosinophil counting. The solid arrow indicates the region of the olfactory cleft where ion concentration measurements were made, in the dorsal recess near sampling area 5. The shaded arrow indicates the general dorsal to ventral transition from Olfactory (OE), Transitional (TE) to Respiratory (RE) epithelia in the mouse nose. Mucus is evident within the lumen (M). Bar = 500 µm. B) Higher power image from panel 1 showing counting regions 1 and 2. Arrows indicate Sirius Red stained eosinophils localized to Lamina Propria (LP) and Respiratory Epithelium (RE). Bar = 100 µm. C) Localization of MBP in the coronal section of a chronic allergen exposed mouse. Primary antibody is mouse anti-MBP and non-specific binding is blocked by mouse IgG incubation. Brown staining indicates MPB (arrow). Regions of Olfactory (OE), Transitional (TE) and Respiratory (RE) epithelia are indicated. Mucus is evident within the lumen (M). Bar = 50 µm. D) Higher power image from panel 3. Arrows indicate MBP localized to Lamina Propria (LP) and Respiratory Epithelium (RE). Olfactory epithelium (OE) is largely devoid of MBP. Mucus (M) does not show any significant non-specific staining. Bar = 50 µm.

**Figure 2:**
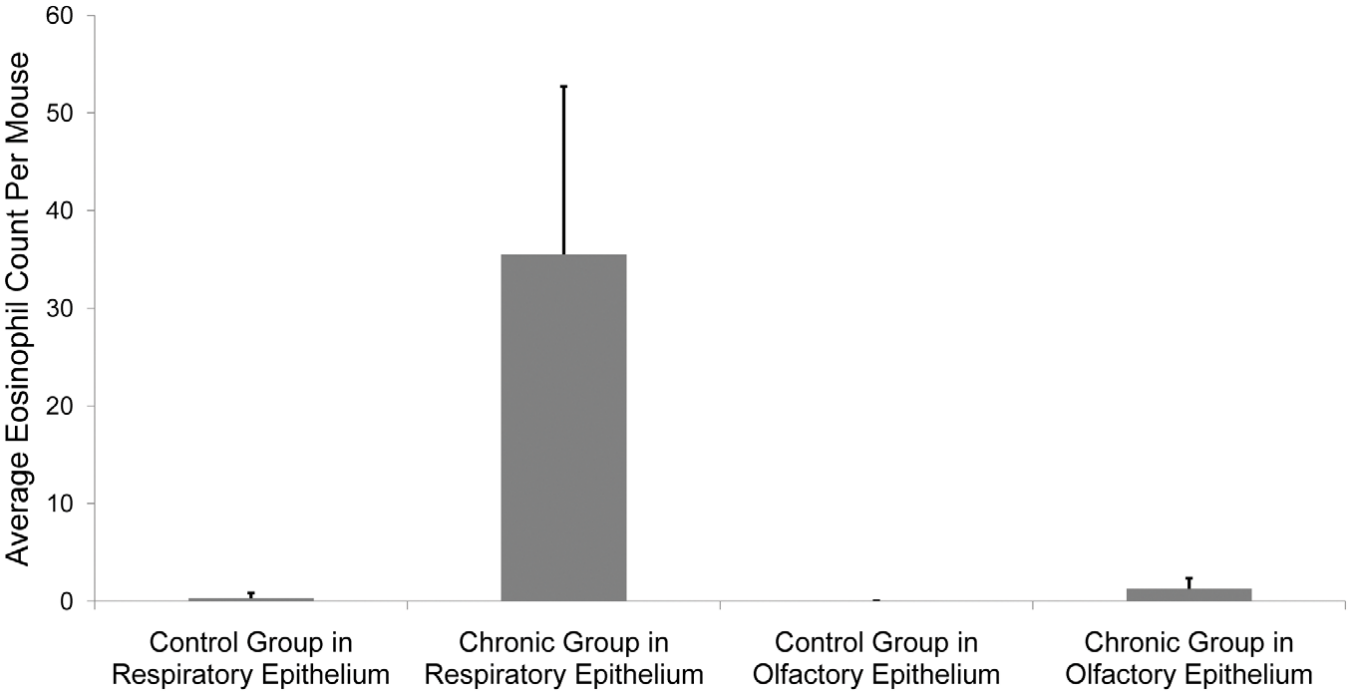
Comparison of eosinophil counts in chronic allergen exposed mice with controls. Average eosinophil infiltration in control and chronic groups. There is a dramatic and significant increase in the number of eosinophils in the respiratory epithelium of the chronically inflamed mice versus the controls, while the olfactory epithelium shows only a modest, yet statistically significant proliferation. Error bars denote standard deviations.

#### Ion concentration measurements

Following surgical removal of the nasal bone, an ion-selective electrode was mounted to a 3D micromanipulator (Narishige MWH3) or to an inchworm motor (Burleigh). The microelectrode was then advanced towards the tissue until the electrode made contact with the surface of the tissue. This contact was identified visually and by the voltage recording. Before penetrating the tissue, the voltage reading was set to 0. Next, the electrode was advanced through the lamina propria and the olfactory epithelium then stopped when the electrical reading disappeared. The loss of the voltage reading indicated that the electrode has passed through the olfactory mucus and has reached the nasal lumen with its tip in the air. To obtain a measurement in the olfactory mucus, the electrode was then retracted until the voltage reading reappeared. Before retracting the electrode entirely through the tissue, a drop of reference solution (Ringers Lactate) with known concentrations of sodium, potassium, and calcium was placed on the tissue. The ion selective electrode was then retracted through the tissue until the tip rested in this reference solution. The latter was verified visually and could also be seen in the voltage readings. Measurements in the reference solution provided the reference point for the concentration calculations. If bleeding occurred during the entry of the microelectrode into the nasal cavity, measurements were terminated and the contralateral side was used instead.

During the measurements the electrode penetrates the tissue and may damage cells resulting in a localized increase in potassium. However, it is unlikely that the small localized insertion damage affects the ion measurements in the mucosa because the volume of a small number of cells is negligible when compared to the volume of the mucus, which was the target for the measurements. Furthermore, if the results are biased by the method, potassium concentration would increase in both treatment groups.

### Effects of Ion Concentration Changes on Human Olfaction

To investigate the physiological significance of our findings on olfaction in humans, participants were enrolled in a translational study to examine the consequences of altering the ionic olfactory microenvironment via nasal irrigation. All study participants gave written, informed consent, and the institutional review board at Northwestern University approved the study.

Participants at least 18 years old were eligible for this study. Individuals who were pregnant, smokers, users of nasal irrigation, users of medications known to interfere with olfactory function, or those with a history of a rhinitis, olfactory dysfunction, or upper respiratory infection in the previous two weeks were excluded from the study [21]–[23]. Age, sex, medications, and co-morbidities were recorded for each test subject. Olfactory threshold was assessed for each participant (see below) before and immediately after rinsing with a solution composed of ion concentrations, which were determined by either the chronically inflamed or the control model. Each of the solutions was tested separately with at least a one-week interval between testing sessions. Though ion concentrations are translated from a mouse model, previous studies have demonstrated similar sodium concentration between the two species, although potassium was found to be higher in humans [13], [24]–[26]. However, there is no consensus in the literature [13].

Irrigation was performed using a commercially available device, the NeilMed squeeze bottle, which employs a user-controlled positive pressure delivery system that allows even non-sophisticated rinsers to perform irrigation with excellent results [27]. All subjects were observed during irrigation to ensure proper technique. Solutions were heated to 37°C and pH-balanced to mimic physiological conditions. The calculated osmolarities of both solutions were very similar (0.20 Osm/L control model, 0.19 Osm/L chronically inflamed model). Both the study participant and test administer were blinded to the contents of the irrigation solution.

Olfactory threshold was determined using the *Sniffin’ Sticks* test. Details regarding the administration and scoring of the *Sniffin’ Sticks* test have been reported elsewhere [21], [22]. In brief, concentrations of n-butanol were established in the *Sniffin’ Sticks* via geometric dilution. The odors were presented to participants by holding pen-like devices 2 cm midline in front of both nostrils. Odor-containing pens were alternated with 2 blanks in a 3 alternative forced-choice model. Triplets of pens are presented in a randomized order to subjects, who are asked to identify the odor-containing pen while blindfolded. Threshold is ultimately scored on a scale of 1–16 (lower scores represent worse olfactory function).

**Table 2:** *In vivo* ion concentrations measured in mice.

	Chronic Rhinitis (N = 8)	Untreated (N = 8)	P-value
Sodium (mM)	84.8±4.45	93.7±3.06	0.04
Potassium (mM)	7.2±0.65	5.7±0.20	0.02
Calcium (mM)	0.50±0.12	0.54±0.06	0.39

Values are expressed as mean ± standard error.

### Statistical Analysis

Averages and standard errors were calculated for the eosinophil counts and the free sodium, potassium, and calcium ion concentration values obtained for the different treatment groups. An analysis of variance (ANOVA) was performed, and if the ANOVA indicated differences among the means, Tukey’s test was used for making pairwise comparisons among the means. Analyses were performed using IGOR (version 6.03, Wavemetrics, Lake Oswego, OR).

Human olfactory threshold scores before and after nasal irrigation within each treatment group in addition to the difference from baseline threshold after irrigation between treatment groups were compared using the nonparametric Wilcoxon signed-rank sum test. With a null hypothesis of no difference in olfaction after nasal irrigation and an alternative hypothesis of a 2 level threshold change in olfaction after nasal irrigation, we calculated that enrollment of 11 patients would provide 0.82 power to show statistical significance for each of the two within-group tests assuming a standard deviation of 2.3 [23]. A two-sided p-value <0.05 was considered statistically significant. Analyses were performed using Stata (version 10.1, StataCorp, College Station, TX).

**Figure 3:**
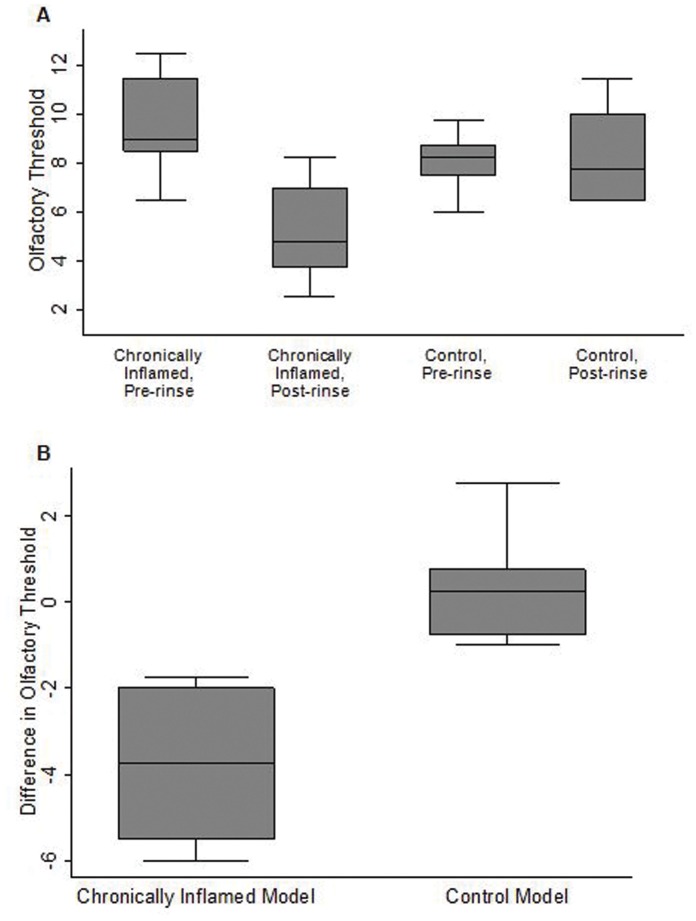
Comparison of olfactory thresholds between treatment groups. A) Boxplots demonstrate that rinsing with the solution replicating ions concentrations found in the chronically inflamed murine model caused a significant elevation in human olfactory threshold compared to rinsing with the solution replicating ion concentrations found in the control murine model (p = 0.003 versus p = 0.75, respectively). There was no significant difference between the baseline olfactory threshold measurements between the treatment groups (p = 0.23). B) The difference in olfactory threshold after rinsing with the solution replicating ion concentrations in the chronically inflamed mouse model was much greater than rinsing with the solution replicating ion concentrations in the control model (p = 0.003).

## Results

### Histology and Immunohistochemistry

Sensitized animals chronically exposed to *A. fumigatus* showed dense inflammatory infiltrates in many areas of the respiratory epithelium and mild inflammation in the olfactory epithelium. Almost all of the inflammatory infiltrates had eosinophilic granules and bi-lobed nuclei, which are typical of murine eosinophils. Quantification of eosinophilic infiltrates in the respiratory and olfactory neuroepithelium was performed in 6 corresponding locations (Fig. 1A and B). Mice chronically exposed to *A. fumigatus* showed significantly greater eosinophil counts in the respiratory epithelium (35.5±5.4) as compared to untreated animals (0.31±0.18, p<0.001). A small, but statistically significant increase of the eosinophil count in the olfactory epithelium of the chronically exposed group was also observed (1.25±0.38) as compared to the untreated group, which showed no eosinophil infiltration in any of the animals (p<0.01). Values are displayed as average ± standard error (Fig. 2).

Staining consistent with specific detection of MBP was evident in chronic allergen exposed sensitized mice (Fig. 1C and D) but not in control animals (not shown). Respiratory and transitional epithelium appeared more heavily stained throughout than olfactory epithelium and the staining in olfactory epithelium was largely confined to the underlying lamina propria and to mucus within the lumen.

### Ion Concentrations

Ion concentrations were measured in 8 control mice and in 8 mice after chronic inflammation was established. Average ion concentrations ± the standard error for sodium, potassium and calcium was 84.8±4.5, 7.2±0.65, and 0.5±0.12 mM in mice with chronic rhinitis, and 93.7±3.06, 5.7±0.2, and 0.54±0.06 mM in control mice, respectively (Table 2). Differences were significant for sodium and potassium concentrations (p<0.05 for both calculations). The corresponding power for the statistics was 0.83 and 0.80 for the sodium and for the potassium concentrations. No significant difference existed in calcium concentration (p  = 0.39).

### Nasal Irrigation

Eleven human participants, free of nasal, sinus, and olfactory disease, were subsequently enrolled to determine the significance of altered cation concentrations in the olfactory mucosa using nasal irrigation. The observed sample median age was 27 (25^th^–75^th^ percentile 23–38) years with a male predominance (64%). None were taking any medications or had co-morbidities known to affect olfactory function.

The median olfactory threshold prior to nasal irrigation was 8.8 (25^th^–75^th^ percentile 7.9–9.5), consistent with previous studies in similar populations [23], [28]. Nasal irrigation with the solution composed of ion concentrations from the chronically inflamed model showed a large elevation of the median olfactory threshold from baseline (9.0 to 4.8, p = 0.003). In contrast, nasal irrigation with the solution composed of ion concentrations from the control model did not significantly change the median olfactory threshold (8.3 to 7.8, p = 0.75). Rinsing with the solution replicating ion concentrations in the chronically inflamed model versus the control model caused a greater elevation in olfactory threshold from baseline (−3.8 versus 0.3, p = 0.003) (Fig. 3).

## Discussion

Our experiments demonstrate that chronic rhinosinusitis in mice alters the cation concentration in the olfactory mucus. We observed a significant decrease in sodium concentration and a marked increase in potassium concentration, while the calcium concentration remained unaffected. We further validated the clinical significance of our findings in human participants using nasal irrigation consisting of ion concentrations from both mouse treatment models in a crossover study. We demonstrated a significant decrease in olfaction when rinsing with ion concentrations found in the chronically inflamed model as compared to the control model. To our knowledge, these measurements provide the first *in vivo* values for ion concentrations in the mucosa of normal and allergic mice as well as the first demonstration of the ability of particular ion concentrations delivered to the olfactory mucosa to impair olfaction in subjects without olfactory dysfunction.

The results of the histology, specifically the vast increase in eosinophilic inflammation in the respiratory epithelium, suggest that *A. fumigatus* is a reliable animal model to mimic allergic rhinitis. Of interest is the slight inflammation observed in the olfactory epithelium. While previous literature has qualitatively observed that inflammation is restricted to the respiratory epithelium, few have actually quantified the results [29], [30]. Our results show that inflammation is slightly more widespread than previously thought. Interestingly, since inflammation was much more apparent in the respiratory epithelium, our findings may also suggest a role for the respiratory epithelium in determining the olfactory mucus cation concentrations.

Eosinophilic inflammation potentially increases nasal secretion and total protein in the mucus [31], [32]. Therefore, the lower sodium ion concentration in the mice with chronic eosinophilic inflammation could have been caused by dilution. However, the unchanged calcium ion concentration and the increased potassium concentration would argue against a strictly hypo-osmotic volume increase. Likewise, any trauma to the epithelium caused by the penetrating electrode would not be responsible for the change in mucosal ionic composition in the chronically exposed cohort, as both groups were subjected to the same electrode penetration.

Instead, the significantly higher potassium ion concentration in the olfactory mucus in the chronic rhinitis group may be the result of epithelial damage produced by chronic eosinophilic inflammation. Eosinophil granules contain several cytotoxic proteins, and eosinophil granule MBP, which is directly toxic to host tissue, including respiratory mucosa [33]. *In vitro*, MPB has been shown to directly damage respiratory and sinus epithelium in a time and dose-dependent manner [34]. A recent study, which examined nasal tissue with its associated mucus from chronic rhinosinusitis patients using immunofluorescence staining for MBP, suggested that the damage of the epithelium occurred from the luminal side [35]. Such epithelial damage presumably releases intracellular potassium ions into the mucus. We demonstrate here that in mice, the olfactory cleft (where the ion concentrations were measured) and the mucus overlying the olfactory mucosa contain MBP and suggest eosinophil degranulation. This is consistent with elevated potassium concentration and the unchanged calcium concentration. The decrease in sodium concentration may be explained by sodium hyperabsorption, a mechanism demonstrated using *in vitro* sinus epithelial cells in chronic sinusitis [36].

Previous studies have examined the olfactory mucus ion concentrations through several methods without much consistency. The results of our ion concentration in the normal state differ from a previous study using a microdialysis technique in mice, which found average nasal liquid [Na+] was 107 mM and [K+] was 8.7 mM [13]. Another study in mice using X-ray microanalysis found [Na+] was approximately 70 mM and [K+] was approximately 20 mM [37]. The reasons for this discrepancy may relate to technique used or site of sampling.

Changes in sodium and potassium ion concentration may result in hyposmia by affecting the gating of OCNCs, key components in the chemo-electrical transduction. It has been shown that these channels are non-specific for monovalent cations. However, the gating by their cyclic nucleotide ligands is altered in the presence of sodium and potassium ions [10]. For example, the apparent affinity for cyclic nucleotide is increased if potassium ions are passing through the channel. Also, in the presence of subsaturating concentrations of the channel ligand, sodium and potassium ions stabilize the open conformation. Interestingly, channel blockers respond differently to the presence of sodium or potassium ions, suggesting that these blockers can sense alterations to the pore secondary to these ions. Together, these results suggest that ions within the pore of the channels influence the response to ligand by changing the stability of the open conformation [10], [11].

An interesting observation is that the potassium concentration in the nasal liquid obtained from humans that suffer from cystic fibrosis is also increased [14]. Patients who suffer from cystic fibrosis often note olfactory dysfunction, suggesting that the increased potassium concentration modulates chemo-electrical transduction.

In the present study, rinsing only with the solution containing higher potassium and lower sodium affected human olfaction, although the duration of olfactory impairment and psychophysical correlations were not assessed. Previous investigation has shown that rinsing with a sodium-citrate containing solution, to bind and reduce free calcium in the nasal mucus, improved olfactory function in patients complaining of hyposmia or anosmia [12]. While it is known that lowering calcium increases OSN firing, our results suggest that the improvement seen in the study may also been due to the effects of sodium in their solution [9].

This study had ample power to detect a 2 level threshold shift in both models, making it unlikely that the insignificant difference in the control model was the result of an underpowered study. Further studies are needed to determine whether both ions are involved in the modulation of olfaction and whether this is dose-dependent. The results also raise the question whether commercially available nasal rinses, which are also composed of various ion concentrations, can alter olfaction.

In summary, we have demonstrated that chronic inflammation alters the ion concentration of the olfactory mucus, which corresponds to olfactory impairment in human participants using nasal irrigation. The determination of free ion concentrations with ion-selective microelectrodes of the olfactory mucus provides reliable and physiologically relevant information about the ionic environment immediately available to the cation channels and receptors involved in the olfactory transduction.
